# High-Fat Diet Alters the Retinal Pigment Epithelium and Choroidal Transcriptome in the Absence of Gut Microbiota

**DOI:** 10.3390/cells11132076

**Published:** 2022-06-30

**Authors:** Jason Xiao, Bingqing Xie, David Dao, Melanie Spedale, Mark D’Souza, Betty Theriault, Seenu M. Hariprasad, Dinanath Sulakhe, Eugene B. Chang, Dimitra Skondra

**Affiliations:** 1 Department of Ophthalmology and Visual Science, University of Chicago, Chicago, IL 60637, USA; jason.xiao@uchospitals.edu (J.X.); david.dao@uchospitals.edu (D.D.); sharipra@bsd.uchicago.edu (S.M.H.); 2 Department of Medicine, University of Chicago, Chicago, IL 60637, USA; bxie@medicine.bsd.uchicago.edu (B.X.); echang@medicine.bsd.uchicago.edu (E.B.C.); 3 Animal Resources Center, University of Chicago, Chicago, IL 60637, USA; mspedale@bsd.uchicago.edu (M.S.); btheriault@bsd.uchicago.edu (B.T.); 4 Duchossois Family Institute, University of Chicago, Chicago, IL 60637, USA; dsouza@bsd.uchicago.edu (M.D.); sulakhe@uchicago.edu (D.S.)

**Keywords:** age-related macular degeneration, high-fat diet, gut microbiome, gut–choroid axis, RNA sequencing, germ-free mice, complement cascade, angiogenesis

## Abstract

Relationships between retinal disease, diet, and the gut microbiome have started to emerge. In particular, high-fat diets (HFDs) are associated with the prevalence and progression of several retinal diseases, including age-related macular degeneration (AMD) and diabetic retinopathy (DR). These effects are thought to be partly mediated by the gut microbiome, which modulates interactions between diet and host homeostasis. Nevertheless, the effects of HFDs on the retina and adjacent retinal pigment epithelium (RPE) and choroid at the transcriptional level, independent of gut microbiota, are not well-understood. In this study, we performed the high-throughput RNA-sequencing of germ-free (GF) mice to explore the transcriptional changes induced by HFD in the RPE/choroid. After filtering and cleaning the data, 649 differentially expressed genes (DEGs) were identified, with 616 genes transcriptionally upregulated and 33 genes downregulated by HFD compared to a normal diet (ND). Enrichment analysis for gene ontology (GO) using the DEGs was performed to analyze over-represented biological processes in the RPE/choroid of GF-HFD mice relative to GF-ND mice. GO analysis revealed the upregulation of processes related to angiogenesis, immune response, and the inflammatory response. Additionally, molecular functions that were altered involved extracellular matrix (ECM) binding, ECM structural constituents, and heparin binding. This study demonstrates novel data showing that HFDs can alter RPE/choroid tissue transcription in the absence of the gut microbiome.

## 1. Introduction

Age-related macular degeneration (AMD) is the leading cause of irreversible blindness in industrialized countries, affecting 196 million individuals worldwide in 2020 [1]. In recent years, growing evidence has indicated that diet and nutrition may play important roles in the pathogenesis of retinal diseases, including AMD and diabetic retinopathy (DR) [2,3,4,5]. Notably, several studies have found significant associations between high-fat diets (HFDs) with increased prevalence and severity of AMD [6,7,8,9]. The retina is extremely metabolically active and requires a unique lipid composition for visual processing, making this ocular tissue highly susceptible to oxidative stress and metabolic fluctuations [10,11,12,13]. Maintaining retinal homeostasis relies heavily on the adjacent retinal pigment epithelium (RPE) and choroid for barrier protection, nutrient supply, lipid transport, and waste clearance [14]. Consequently, RPE/choroid pathology often precedes signs of retinal dysfunction and may be particularly sensitive to physiologic changes induced by HFDs [14,15,16]. Several mechanisms have been proposed as to how HFDs can reproduce or accelerate retinal disease: fatty acid signaling, metabolic dysregulation, vascular remodeling, and persistent inflammation [15,17,18]. Crucially, HFDs may disrupt the complex equilibrium between the RPE/choroid and retina, subsequently inducing hallmark features of AMD, including the deposition of lipids and proteins beneath the RPE, the thickening of Bruch’s membrane (BM), and choroidal neovascularization (CNV) [16,19,20,21,22,23].

Emerging literature suggests that HFD-induced features of AMD may partially be facilitated by the gut microbiome. Trillions of gut microbes function in diverse ways to impact health homeostasis, including the regulation of inflammation and metabolic signaling [24]. Beyond the gastrointestinal tract, the effects of gut microbiota–host interplay are observed in distant anatomic organs, including the heart, lungs, and brain [25,26,27]. Although research on retinal disease and the gut microbiome is in its nascent stage, several studies have identified strong associations with the intestinal microbiome and AMD [28,29,30]. For example, Andriessen et al. demonstrated that HFDs can induce gut dysbiosis, which in turn exacerbates laser-induced CNV in a model of AMD [31]. Other studies have reported HFD-induced changes to gut microbiota composition and function, suggesting diet and gut microbiota are closely related [32,33].

Considering this, a connection may exist between the gut microbiome, HFDs, and the retina, as well as the RPE/choroid. We recently demonstrated that retinal transcription in germ-free (GF) mice can be altered by an 8-week course of HFD [34]. However, whether and how HFDs affect the adjacent RPE/choroid, which is essential for retinal homeostasis, independent of the gut microbiome, is still unknown. In this study, we investigated changes in RPE/choroid transcriptome induced by HFDs using high-throughput RNA-sequencing in GF mice. We used the framework of AMD pathogenesis to highlight key genes and pathways in the RPE/choroid corresponding to AMD, such as inflammation and angiogenesis, which were significantly altered in response to HFDs in the absence of the microbiome.

## 2. Materials and Methods

### 2.1. Animals and Diets

Mouse experiments were approved by the University of Chicago Institutional Animal Care and Use Committee and adhered to research guidelines established by the Association for Research in Vision and Ophthalmology (ARVO). Germ-free (GF) C57BL/6 adult, male mice were bred and housed in the Gnotobiotic Research Animal Facility at the University of Chicago. Starting at 7 weeks of age, GF mice were placed on a normal diet (ND) or high-fat diet (HFD) for 8 consecutive weeks. The HFD (Teklad Custom Diet TD.130135) consisted of 44.9% saturated fat, 14.8% protein, and 40.3% carbohydrate by caloric content (Envigo, Indianapolis, IN, USA). Of the 40.3% carbohydrates, approximately 21% came from sucrose. The normal diet (ND) consisted of 12% fat, 22% protein, and 66% carbohydrate, with an estimated 0.3% derived from sucrose (Envigo, Indianapolis, IN, USA). GF mice lived under a 12-h light cycle, and environmental conditions such as temperature and humidity were in accordance with The Guide for the Care and Use of Laboratory Animals, 8th edition [35]. At 15 weeks of age, mice were euthanized with carbon dioxide and cervical dislocation. RPE/choroid samples were placed on ice and processed for RNA-sequencing.

### 2.2. Sterility Monitoring

To ensure sterility, GF mice were housed in positive-pressure incubators and fed diets that had been irradiated and autoclaved (250 °F for 30 min). Germ-free status was evaluated as described previously [36]. Briefly, fecal samples were collected every week and cultured aerobically at 37 °C and 42 °C, as well as anaerobically at 37 °C. Cultures were assessed after 1, 2, 3, and 5 days. No positive cultures were identified throughout the study. Additionally, DNA extraction and quantitative real-time polymerase chain reaction (RT-PCR) were performed on fecal samples to screen for contamination using bacterial primers for the 16 S RNA-encoding gene (IDT, 8 F was 5′-AGA GTT TGA TCC TGG CTC AG-3′, and 338 R was 5′-TGC TGC CTC CCG TAG GAG T-3′).

### 2.3. RNA Extraction

Eyes were enucleated, and RPE/choroid tissue was isolated on ice with all equipment, surfaces, and tubes treated with RNase decontamination solution (Thermo Fisher Scientific, Waltham, MA, USA) prior to use. Samples were stored at −80 °C in RNAlater solution (Thermo Fisher Scientific, Waltham, MA, USA) until RNA was extracted using the RNeasy kit from Qiagen (Qiagen, Hilden, Germany). Concentrations were quantified using a Nanodrop 2000c (Thermo Fisher Scientific, Waltham, MA, USA) prior to sequencing.

### 2.4. RNA Sequencing

RNA from eight samples from GF-ND (*n* = 4) and GF-HFD (*n* = 4) was used for analysis. A Bioanalyzer at the University of Chicago Genomics Core was used to determine that RNA quality met appropriate RNA integrity numbers (RIN). Next, cDNA libraries were constructed using TruSeq RNA Sample Prep kits (Illumina, San Diego, CA, USA) to generate 100bp paired-end reads, which were indexed for multiplexing and sequenced using PE100bp on the NovaSeq 6000 System (Illumina, San Diego, CA, USA). Data was provided in FASTQ format and analyzed in R.

### 2.5. Statistical Analysis

The secondary analysis of RNA-sequencing data was conducted in Globus Genomics, an enhanced, cloud-based analytical platform that provides Next-Generation Sequence analysis tools and workflow capabilities. Tools such as STAR [37], featureCounts [38], and Limma [39,40] were run from within the Globus Genomics platform. We used STAR (version 2.4.2 a, Stanford University, Stanford, CA, USA) aligner default parameters to align the RNA-sequencing reads to the reference mouse genome (GRCm38) for all samples. A raw gene expression count matrix was generated with featureCounts (version subread-1.4.6-p1), and gene annotation was acquired from Gencode vM23 [41]. The STAR default parameter for the maximum mismatches was 10, which was optimized based on mammalian genomes and recent RNA-sequencing data.

Genes with low expression (count-per-million < 10) were filtered using edgeR [42,43]. Significant DEGs estimated by Limma with an adjusted *p*-value < 0.05 and LogFC > 1.5 were selected for further downstream analysis. Enrichment analysis in Lynx suite took both the upregulated and downregulated DEGs in GF and extracted the over-represented gene ontology functional classification (molecular functions, biological processes, and cellular components) [44]. The enrichment gene ratio was measured using the number of input DEGs that mapped to the pathway divided by the total number of genes in that pathway. A list of all DEGs is available in Supplementary Table S1.

## 3. Results

### 3.1. High-Fat Diet Is Associated with Changes in the Rpe/Choroid Transcriptome

**Figure 1:**
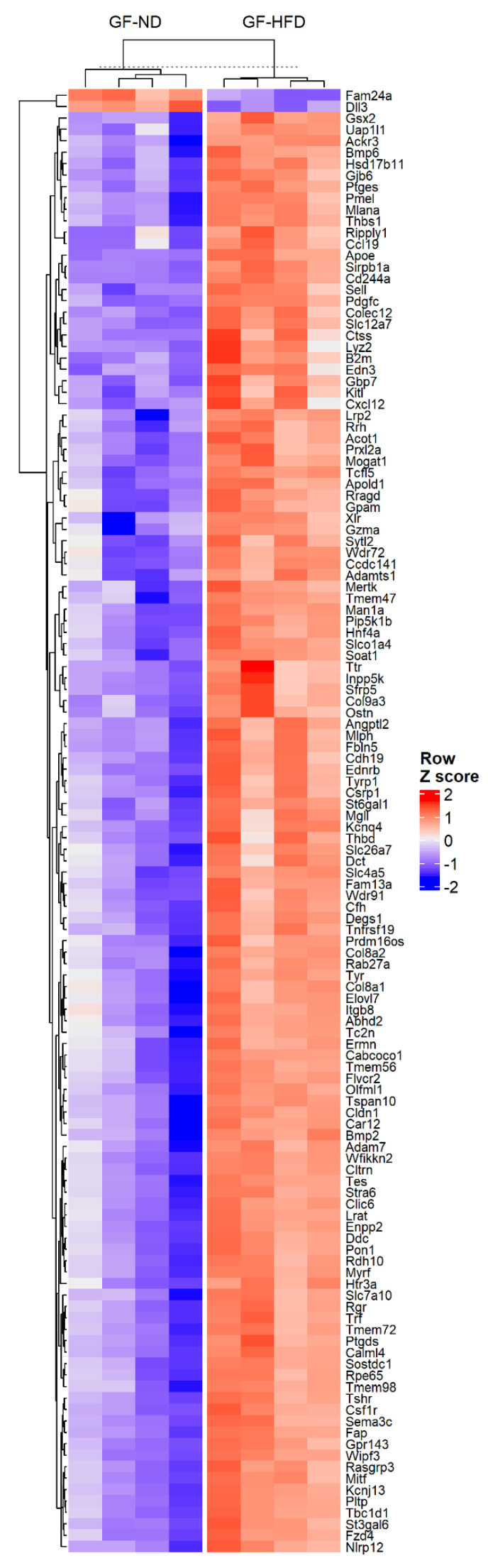
Heatmap with hierarchal clustering demonstrating DEGs with |LogFC| > 1.5 and adjusted *p*-value < 0.01 between germ-free mice on normal diet (GF-ND, *n* = 4) and germ-free mice on high-fat diet (GF-HFD, *n* = 4). Z-score was calculated using LogFC values, with red and blue colors indicating upregulated and downregulated genes, respectively.

In order to study the effects of a HFD on the RPE/choroid in the absence of the microbiome, we performed high-throughput RNA-sequencing (RNA-seq) on RPE/choroid tissue in GF-ND and GF-HFD mice (*n* = 4 per group). After the removal of pseudogenes and uncharacterized cDNA using the National Center for Biotechnology Information (NCBI) database and applying a false discovery rate (FDR) < 0.05 with an absolute LogFC > 1.5 threshold, 649 differentially expressed genes (DEGs) were identified for downstream analysis. Of the 649 DEGs identified, the majority of the genes were transcriptionally upregulated by HFD, with only 33 genes downregulated. A list of all DEGs is available in Supplementary Table S1. The top 30 overexpressed genes are shown in Table 1, which include genes involved in natural killer (NK) T-cell functioning, such as Natural killer cell receptor 2B4 (*Cd244a*) and Natural cytotoxicity triggering receptor 1 (*Ncr1*), as well as inflammatory markers, including Tumor necrosis factor receptor superfamily member 13B (*Tnfrsf13b*), C-C motif chemokine ligand 19 (*Ccl19*), and Prostaglandin-endoperoxide synthase 2 (*Ptgs2* or *Cox-2*). The top 30 overexpressed genes also included mediators of endothelial adhesion and vessel permeability, notably Selectin E (*Sele*) and Lysophosphatidic acid receptor 3 (*Lpar3*). Additionally, a heatmap was generated using an FDR < 0.01 and absolute LogFC > 1.5 threshold to demonstrate the hierarchical clustering of the DEGs by experimental groups, indicating that the changes observed in RPE/choroid transcription likely could be attributed to HFD in the absence of microbiota (Figure 1).

### 3.2. High-Fat Diet Upregulates Multiple Biological Processes and Genes Related to Inflammation and Angiogenesis

**Figure 2:**
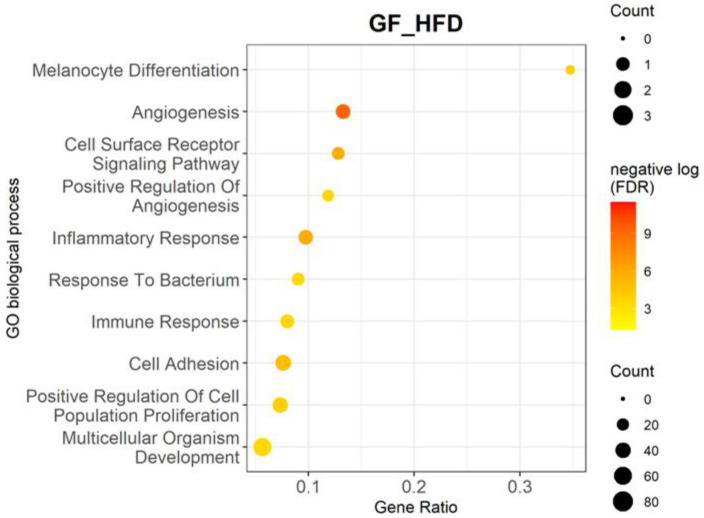
Enrichment analysis of DEGs between GF-HFD mice (*n* = 4) and GF-ND mice (*n* = 4) using Lynx. Gene ontology analysis is shown for top 10 biological processes upregulated in GF-HFD mice compared to GF-ND mice, highlighting pathways including the angiogenic, inflammatory, and immune responses. The corresponding table demonstrates detailed statistics and genes involved in these processes.

After identifying 649 DEGs, we performed enrichment analysis using the Lynx suite for gene ontology (GO) in order to identify over-represented biological processes in the RPE/choroid of GF-HFD mice relative to GF-ND mice [44]. This unbiased approach identified angiogenesis as the most significantly upregulated biological process (adjusted *p*-value = 5.51 × 10^−10^) due to HFD intervention (Table 2, Figure 2). Among the angiogenic genes affected were Vascular endothelial growth factor C (*Vegfc*); angiopoietin genes *Angpt1*, *Angpt2*, and *Angptl2*; their respective receptors *Tie1* and *Tie2* (*Tek*); and platelet derived growth factors *Pdgfc* and *Pdgfd*, which together are involved in angiogenesis, maturation, and vascular remodeling. Additionally, the GF-HFD group demonstrated upregulation of GO biological processes related to the inflammatory response and immune response, such as Complement factor H (*Cfh*) and TNF superfamily genes *Tnfaip2*, *Tnfrsf11b*, *Tnfrsf13b*, *Tnfrsf19*, *Tnfrsf1b*, *Tnfsf10*, and *Tnfsf14*.

**Figure 3:**
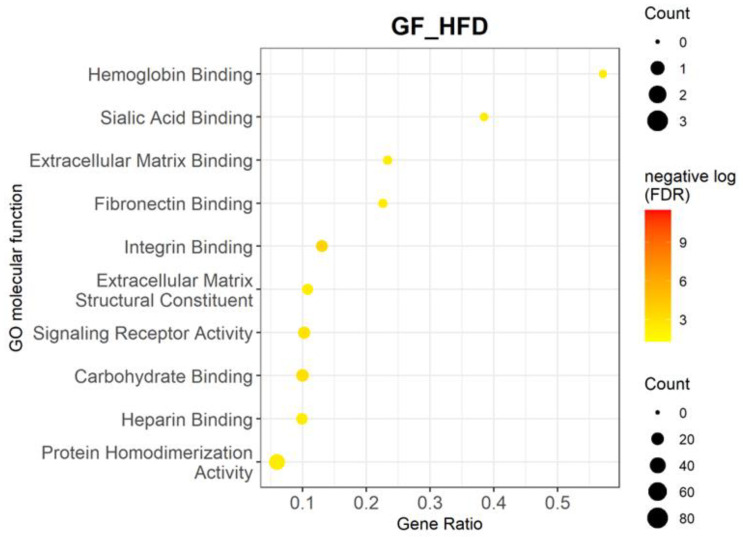
Enrichment analysis of DEGs between GF-HFD mice (*n* = 4) and GF-ND mice (*n* = 4) using Lynx. Gene ontology analysis is shown for top 10 molecular functions upregulated in GF-HFD mice compared to GF-ND mice. Functions such as extracellular matrix (ECM) binding, ECM structural constituents, and heparin binding are highlighted. The corresponding table demonstrates detailed statistics and genes involved in these functions.

Our analysis also indicated significant alterations induced by HFD in molecular processes such as extracellular matrix (ECM) binding, ECM structural constituents, and heparin binding (Table 3, Figure 3). As the RPE/choroid has multiple functions in addition to providing nutrient exchange to the retina, the connective tissue cells types such as fibroblasts, melanocytes, pericytes, and immune cells are active players in maintaining homeostasis [45]. Genes involved in ECM interactions that were upregulated include Apolipoprotein E (*Apoe*)*;* the matrix metalloproteinase *Adamts9*; collagens *Col2a1*, *Col8a1*, and *Col10a1*; and fibronectins such as *Fbln1* and *Fbln5*.

## 4. Discussion

Diet and nutrition are significant risk factors in retinal disease pathobiology, including AMD and DR [46,47]. Specifically, several studies have emphasized an association between HFDs and the increased prevalence and progression of AMD [8,9]. Using animal models of neovascular AMD, our team previously has demonstrated that HFDs can increase laser-induced CNV lesion size, vascular leakage, and the formation of sub-RPE deposits [19]. Other studies, too, have recapitulated AMD-like features in mice fed HFDs [22,23]. Given the role of the gut microbiome in immunomodulation, nutrition, and energy metabolism, there is a growing body of literature connecting the gut microbiome and AMD [2,29,30,48]. Gut microbiota may be key mediators of HFDs in retinal disease, whereby HFDs induce gut dysbiosis, increase intestinal permeability, and induce chronic inflammation in AMD models irrespective of total energy intake [31]. We previously studied HFD-induced changes in retinal transcription independent of gut microbiota; however, its impact at the level of the RPE/choroid is unknown [34].

In this study, we sought to uncouple the impact of HFDs on RPE/choroidal biology from the gut microbiome. In both dry and wet AMD, pathological changes typically occur first in the supporting tissue of the RPE/choroid before damage is observed to underlying retinal cells [14]. In addition, there is evidence in the RPE/choroid of lipid accumulation that contain fats derived exclusively from diet, which helps corroborate the role of diet in RPE/choroid biology [49]. To the best of our knowledge, this is the first study to explore the transcriptional changes induced by HFDs in the RPE/choroid in the absence of the gut microbiome by using germ-free mice. After analyzing and filtering the data, we identified 649 DEGs and performed GO enrichment analysis to highlight changes in important biological pathways.

### 4.1. High-Fat Diet Affects Gene Expression in Angiogenic Pathways in Germ-Free Mice

Angiogenesis is a hallmark feature of wet AMD, which may account for up to 90% of cases of AMD-related severe vision loss [50]. During this process, abnormal blood vessels from the choroid infiltrate the sub-RPE space, resulting in vascular leakage, bleeding, scarring, and damage to the macula [51]. Consequently, therapeutics that limit angiogenesis are widely-used to delay AMD progression, such as anti-VEGF therapy [52]. In the absence of the microbiome, HFD upregulated pathways are involved in angiogenesis, as well as in its positive regulation (Figure 1). In addition to *Vegfc*, HFD significantly elevated the angiopoietin genes *Angpt1*, *Angpt2*, and *Angptl2*. Angiopoietins are selective growth factors for vascular endothelium. The ANG signaling pathway is heavily involved in vascular development in both the choroid and retina [53]. Specifically, choroidal neovascularization occurs when Vegf and Angpt2 are elevated in conjunction with the disruption of Bruch’s membrane and the RPE [51]. This relationship is substantiated by other studies showing that Angpt2 is necessary for ischemia-induced neovascularization in mice lacking Angpt2 [53]. Recently, the FDA approved faricimab, a biologic targeting both VEGF and ANGPT2, in the treatment of AMD and DR. We also detected the concomitant upregulation of the respective angiopoietin receptors, *Tie1* and *Tek* (*Tie2*), which are tyrosine kinases that transduce the signaling pathways for vessel maturation [54]. VEGF and ANG drive complementary angiogenic pathways, with VEGF inducing early vessel sprouting and growth, whereas ANG1 mediates vascular remodeling, maturation, and protection [55,56]. Similarly, angiopoietin-like 2 protein (Angptl2) is involved in angiogenesis and vasculogenesis [57]. Studies have found that excess ANGPTL2 signaling results in chronic inflammation and irreversible tissue remodeling [58]. Consequently, VEGF-inhibition may be only effective so long as neovascularization is in its nascent stage, which may explain why an estimated nearly 50% of patients with neovascular eye disease do not respond to anti-VEGF treatments such as bevacizumab or ranibizumab [53,59,60,61]. Clinical trials have begun that either target ANG2 alone or concurrently with VEGF for treatment of AMD [62,63].

In addition to the ANG pathway, we found that HFDs elevated the transcription of Platelet-derived growth factors C and D (*Pdgfc* and *Pdgfd*), which are angiogenic factors that play critical roles in several ocular neovascular diseases, including AMD [64]. PDGF levels are normally low or undetectable, but they become elevated in numerous vascular and cardiovascular pathologies [65]. Crucially, PDGF ligands help recruit and maintain choroidal fibroblasts and pericytes, which serve as scaffolds for vascular endothelium. In mouse models of wet AMD, both Pdgfc and Pdgfd expression is upregulated. PDGFC also positively regulates other pro-angiogenic factors, such as VEGF and PDGFB [64]. PDGFs are targets for AMD treatment both in preclinical trials and in clinical trials, where dual anti-PDGF and anti-VEGF therapy has demonstrated superior efficacy to anti-VEGF monotherapy [66,67].

Additional genes involved in angiogenesis that were transcriptionally upregulated with HFD included *Lpar3* and *Tnfaip2*, as well as *Cyp1b1* and *Cxcr3*. Previous studies have linked Cxcr3 dysregulation with wet AMD, though the nature of this pathway is not clearly understood [68]. *Cyp1b1* is another gene required for the neovascular response to ischemic retinopathy as it plays roles in angiogenesis and capillary morphogenesis [69,70].

### 4.2. High-Fat Diet Alters Gene Expression Involved in Inflammatory and Immune Response Pathways in Germ-Free Mice

Inflammation is present during every stage of AMD pathology, beginning with drusen formation; drusen are deposits of cellular debris that serve as nodes for inflammatory processes [71]. Inflammatory signaling molecules, macrophages, and activated resident microglial cells localize to sites of drusen deposits, Bruch’s membrane degeneration, and CNV [72,73]. Serum levels of inflammatory markers such as C-reactive protein (CRP), as well as retinal autoantibodies, are significantly associated with AMD advancement [74,75]. As a result, there are a host of preclinical and clinical trials using anti-inflammatory agents to treat AMD [72,76]. Current literature supports the notion that HFDs can promote the pathogenesis of diseases in multiple organ sites by means of inducing chronic, low-grade inflammation and accelerating age-related cellular processes [77]. Part of this “inflammaging” is thought to be mediated by gut microbiota that can release inflammatory products, signal to other organ sites, and regulate circadian rhythm [78,79].

In this study, we found that HFDs in the absence of the microbiome altered the gene transcription of inflammatory pathways in the RPE/choroid. One of the top 10 biological processes upregulated by HFDs was the inflammatory response (Figure 1). Transcriptional upregulation was observed in multiple genes involved in the chemoattraction, activation, and functioning of natural killer T (NKT) cells, such as *Cd244a*, *Cd48*, *Cxcl10*, Granzyme A (*Gzma*), Perforin (*Prf1*), and *Il12b*. NKT cells act at the interface of innate and adaptive immunity, acting rapidly to immunogenic stimuli and possessing powerful cytotoxic capabilities [80]. NKT cell activity has been associated with a number of neurodegenerative/neuroinflammatory diseases such as Alzheimer’s and multiple sclerosis [81,82]. Immunohistological studies have identified several immune cell-types in the subretinal space in AMD patients, including natural killer lymphocytes, suggesting that NKT cells may play a role in its pathogenesis [83]. NKT cells are reported to accumulate during laser-induced CNV. In support of this, two different NKT-deficient mouse strains demonstrated decreased CNV severity and *Vegf* expression. Furthermore, co-culturing RPE with NKT cells confirmed the ability of NKT cells to produce VEGF, potentially driving further angiogenesis [84]. Haplotypes of killer cell immunoglobulin-like receptors (KIRs) found on NKT cells are associated with AMD in certain populations [85].

*Cd244a*, a risk factor for inflammatory diseases such as rheumatoid arthritis, was the most highly upregulated gene in our data set [86]. Cd244a is a cell-surface receptor on NKT cells that mediates their expansion, activation, and cytotoxicity [87]. In addition, its principal binding partner, *Cd48*, was also upregulated in GF mice fed a HFD. Cd48 is also involved in other immunoregulatory functions, including immune cell adhesion and the co-stimulation of antigen-presenting cells [88]. *Gzma* and *Prf1*, which encode proteins underlying the main mechanisms by which NKT cells induce cytotoxicity, were also upregulated by HFDs [89]. The additional upregulated genes included *Cxcl10*, a activator and recruiter of NKT cells, and *Il12b*, a cytokine that serves as a growth factor for NKT cells, enhances their cytolytic activity, and induces interferon-gamma production [90,91]. Several studies have identified IL-12 as a potential driver of chronic inflammation in the context of AMD [92,93]. In addition to NKT cells, T cell-related genes such as *Ccl19*, *Ccl22*, and *Cd28* were upregulated, which are involved in activation and chemoattraction [94].

Several other inflammatory genes upregulated included *Ccl4*, which has been shown to mediate inflammation in response to retinal damage, as well as prostaglandin and TNF families [95]. Tumor necrosis factors (TNF), particularly TNF-alpha, are pro-inflammatory cytokines whose signaling is thought to play a role in the neovascularization of the RPE/choroid and AMD pathogenesis [96,97,98]. Many members of the TNF superfamily were upregulated in the GF-HFD group, including *Tnfaip2*, *Tnfrsf11b*, *Tnfrsf13b*, Tnfrsf1b, *Tnfsf10*, and *Tnfsf14*. Genetic variations of *Tnfsf10* in particular have been associated with AMD [99]. Anti-TNF therapies may be effective in treating AMD and reducing the frequency of anti-VEGF therapy [100,101]. Finally, we noted the elevation of Prostaglandins D, E, and I (*Ptgds*, *Ptges*, and *Ptgis*, respectively), along with *Cox-1* and *Cox-2* (*Ptgs1* and *Ptgs2*), which synthesize prostaglandins. Prostaglandins regulate vascular permeability and vasodilation and are induced in the inflammatory response. Elevated transcripts of these genes may contribute to the bridge between inflammation and aberrant blood flow and vascular leakage; however, the role of prostaglandins in the retina and RPE/choroid is unclear, with only sparse evidence suggesting prostaglandins are implicated in the pathogenesis of AMD and DR [102]. In a rat model of CNV, ketorolac, a type of anti-inflammatory NSAID that inhibits COX enzymes, was shown to significantly reduce CNV leakage and vascular budding [103].

### 4.3. High-Fat Diet Affects Gene Expression Involved in the Complement System

One specific immune pathway that is highly implicated in AMD is the complement system, with existing reviews that detail how complement activation may influence AMD pathogenesis [104]. The complement system is important for the removal of immune complexes, apoptotic cells, and adaptive immunity. With over 40 proteins involved in the cascade, alterations at multiple steps can impart significant differences in the overall inflammatory response. In our transcriptional analysis, we identified several DEGs involved in the complement cascade, such as *C1qb*, *C2*, *C4b*, and *Cfh*. CFH was the first complement protein associated with AMD in genetic studies and can be directly synthesized by RPE cells [105,106,107]. The CFH Y402H polymorphism is thought to increase AMD risk by up to sevenfold [108]. CFH binds to polyanionic moieties, in particular sulfated glycosaminoglcyans (GAGs). The CFH Y402H polymorphism is thought to alter the binding property of CFH to GAGs, such as heparan sulfate [109]. Interestingly, one of the top 10 molecular functions upregulated by HFD was heparin binding, which is a well-established mechanistic function of CFH (Figure 2) [110]. In addition to CFH, variations in C2 have been linked to differential risk for AMD by several groups [111,112]. We also identified elevated expression of *C4b*, whose variants may also play a role in AMD [99].

While the precise role of different components of the complement system in AMD pathology is unclear, it appears that the RPE/choroid is an important hub for complement activity. The membrane attack complex (MAC) is the endproduct of the complement cascade, and studies report MAC localization to the choriocapillaris of the choroid, as opposed to RPE or retinal tissue [113,114]. After comparing the presence of MAC in aged human tissues across multiple organs, Chirco et al. determined that the MAC selectively accumulates in the choroid, which may partly explain the tight association between AMD and the complement system [115]. In vitro studies confirm that direct exposure of the MAC to choroidal endothelial cells promoted death and upregulated pro-angiogenic factors, ultimately leading to CNV [116]. HFDs have been shown, in addition to promoting chronic inflammation, to specifically induce complement activation in animal models and upregulate levels in the blood [117]. One study found that aged *Cfh*^+/−^ mice fed a high-fat, high-cholesterol diet developed features of AMD, including complement dysregulation, sub-RPE deposits, and impaired visual function changes resulting from changes in RPE morphology [118]. Further studies looking into the interactions between age, diet, and complement dysregulation are required. A number of targets in the complement cascade are being targeted for the potential therapeutic benefit of AMD [119,120].

### 4.4. Additional Genes and Pathways Are Differentially Represented by High-Fat Diet in Germ-Free Mice

Several other pathways that were affected by HFD were related to ECM interactions and RPE function. Changes in the ECM, including sub-RPE deposits and the thickening of Bruch’s membrane, are often the initial clinical symptoms of AMD. These deposits consist of many different substances, including ECM proteins, complement, lipids, and other cellular debris. GF-HFD mice demonstrated elevated transcriptional levels in various ECM components, such as *Apoe*, *Adamts9*, *Esm1*, *Col2a1*, *Col8a1*, *Col10a1*, *Fbln1*, and *Fbln5*. As a key player in lipid, vitamin, and cholesterol transport, variants in *APOE* are found to be associated with AMD [121]. In AMD, unbalanced lipid exchange may drive RPE decline and impair the exchange of lipids across from the choroid, which may potentiate pathological changes in the eye [122]. APOE is the most abundant lipoprotein component of drusen, which can stress the RPE, hinder nutrient exchange, and serve as a focal point for generating inflammation and CNV [108,123]. Additionally, APOE has been found to interact with complement, co-localizing with the MAC in human eyes [124]. Within the apolipoprotein family, *Apold1* and *Apol7c* also were identified DEGs in GF-HFD mice. *Apold1* (Apolipoprotein L Domain Containing 1) is a protein that regulates endothelial and vascular functioning. Its expression has been found to be elevated in the RPE/choroid of AMD patients compared to controls [125].

Our analysis also identified the elevated expression of other matrix proteins, including collagens, metalloproteinases, and other secreted proteins. Of note, collagen *Col8a1* and *Col10a1* expression increased, with both genes having genetic associations with AMD [126]. In addition, *Col2a1* previously has been linked with ocular disorders such as retinal detachment [127]. Fibulins *Fbln1* and *Fbln5* are differentially expressed ECM glycoproteins, and of note, one study has associated mutations in *FBLN5* with AMD [128]. *Adamts9* is a metalloproteinase that was identified by the AMD Gene Consortium as one of seven new loci associated with AMD [99]. Endothelial cell-specific molecule 1 (*Esm1*) is a secreted protein that is a highly overexpressed gene in oxygen-induced retinopathy. Esm1 modulates Vegf bioavailability and leukocyte extravastion [129]. In addition, *ESM1* variants are associated with increased levels of advanced glycation products (AGEs), which are significant components of macula drusen and basal lamina deposits [130,131]. Patients fed a high-fat diet had elevations in blood levels of AGEs [130].

Given the functional relevance and spatial proximity, the RPE is thought to be the principal driver of debris accumulation and deposition in both aging and AMD eyes [108,132]. A HFD altered the transcription of multiple genes related to RPE function and the visual cycle, including *Lrat*, *Rpe65*, *Rdh5*, and *Rdh10*. These genes were also found to be upregulated in aged, human RPE [133]. LRAT is an enzyme that catalyzes vitamin A esterification into all-*trans*-retinyl esters, which are converted to 11-*cis*-retinol via RPE65 [134]. By-products of the RPE visual cycle are common constituents of lipofuscin, which are toxic to RPE cells and may impact AMD pathogenesis [135]. These by-products positively regulate LRAT expression, and thus a positive feedback mechanism has been suggested that links visual cycle by-product accumulation with the RPE visual cycle [133]. HFD-fed mice tend to have thicker Bruch’s membranes, RPE dysfunction, and greater accumulation of basal laminar deposits, suggesting HFDs may alter the ECM in AMD pathobiology [22,23].

Across HFD research, the composition of HFDs in animal studies ranges greatly, spanning from 8.3–80% fat content [136]. The selection of HFD, therefore, deserves further discussion. The HFD used in these experiments approximates a “Western diet”, which, according to reports from the National Health and Nutrition Examination Survey (NHANES), is around 35% fat by energy content and 23% by simple carbohydrates such as sucrose [137,138]. Whereas diet duration did not vary, the composition was chosen to parallel human dietary patterns and thus included a high sucrose component of around 21% by energy content. Additionally, we wanted to examine how our results compared to other HFD RPE/choroid transcription data in mice with intact gut microbiota. In this context, Andriessen et al. detected elevated levels of mRNA of *Il6*, *Tnf*, and *Vegfa* in the choroids of mice fed HFD compared to mice fed a regular-chow diet, suggesting an increase in endotoxemia and systemic inflammation [31]. Separately, Zhang and colleagues demonstrated increased *Vegfa*, as well as a non-significant rise in *ApoE* mRNA in the RPE/retina in mice fed HFD [22]. That Andriessen et al. and Zhang et al. demonstrated elevated *Vegfa* expression, in conjunction with our GF model of elevated *Vegfc*, suggests the HFD may impact angiogenesis with or without microbiota [22,31]. Additionally, our experiments with GF mice detected the relative overexpression of numerous *Tnf* superfamily genes and *ApoE*, which were also implicated in these other studies. Nevertheless, due to study design differences with respect to HFD composition and the mode of gut microbiota depletion, it is difficult to directly compare these results.

Within our model, when contrasting these results with previously identified changes in retinal transcription of GF mice, HFDs induced a greater shift in transcription in the RPE/choroid, with little overlap in the genes affected. In fact, the only two overlapping genes were *Ms4a6b* (membrane spanning 4-domains A6A) and *Hbb-bs* (hemoglobin subunit beta), both of which were upregulated in the retina and in the RPE/choroid [34]. These transcriptional differences may partly be due to the distinct functions of the retina and RPE/choroid within the visual system, with the RPE/choroid providing barrier protection, nutrients, substrates, and waste clearance for the neural retina [14]. Additionally, the separate cell types and lack of vascular barrier of the choroid compared to the immune-privileged retina could further contribute to discrepancies in transcriptional effects caused by systemic, metabolic changes induced by diet in the absence of gut microbiome. As changes in RPE/choroid biology often precede overt signs of retinal dysfunction, these findings also suggest tissue-specific sensitivity and a response to dietary changes over a fixed duration. One recent study found that mice fed a HFD with 45% fat for 12 months demonstrated no retinal function abnormalities as measured by ERG; however, evidence of lipid deposition, RPE distortion, and endothelial vacuolization was present in the HFD-fed group [139]. Interestingly, an unbiased pathway analysis of transcriptional changes in the retina and RPE/choroid in GF-HFD mice both point toward alterations in angiogenic and inflammatory pathways in response to the dietary intervention. Overall, however, these results indicate that the diet-microbiome-transcriptome interactions could be distinct in the RPE/choroid compared to the retina. Because these experiments were performed using GF mice, the transcriptional pathways affected by HFD in the RPE/choroid could either be attenuated or exacerbated by the presence of the gut microbiome. Gut microbiota can significantly change dietary metabolism and the resultant small molecule profiles of the host organism, potentially influencing the transcriptional responses of the retina and RPE/choroid. Likewise, HFDs have the capacity to alter the gut microbiome, a process that itself may affect retinal health and homeostasis. As a result, the microbiome-dependent and microbiome-independent effects of HFD on the RPE/choroidal transcriptome are complex and require further investigation.

While the pathobiology between HFDs and retinal disease is studied mostly in rodent models, its connection with human pathways and disease still holds biological plausibility, though proof of causality is absent. Nevertheless, there are numerous epidemiologic associations of AMD with high-fat or Western diets, with some reporting up to a threefold greater incidence of late AMD [9,140]. In addition to low-grade inflammation caused by HFDs, its potential role in aberrant lipid homeostasis may further contribute to AMD pathology, especially given that about 40% of drusen is composed of lipid-containing particles [141]. The genetic components of lipid metabolism and transport, such as *APOE* alleles, along with serum lipid levels, have also been identified as differentiators for AMD risk [142,143]. Beyond AMD, saturated fatty acid intake has been linked with increased prevalence and severity of DR, with some studies reporting an odds ratio of 2.37 [5,144]. In rhesus monkeys, which are more biologically similar to humans, a high-fat Western diet for 2–4 years resulted in retinal changes characteristic of human DR, including venous engorgement, macular exudates, and hemorrhages [145]. Further studies are warranted to bridge HFD animal studies with human manifestations of AMD and DR.

## 5. Conclusions and Limitations

This study presents data that suggests diet can impact the RPE/choroid transcriptome in the absence of gut microbiota. We use the framework of AMD pathogenesis to highlight gene expression and biological pathways that contrast greatly between GF-HFD and GF-ND mice. In particular, HFDs altered the transcription of genes involved in angiogenesis, inflammation, complement, and RPE function. As an unbiased exploration of the RPE/choroid transcriptome, the major limitation of this study is its reliance on RNA-sequencing. Future studies should include the quantitative PCR of specific genes, protein expression/proteomics, and functional assays in order to better elucidate the putative role of HFDs in RPE/choroid biology. In this current investigation, we did not address the effects of HFD-induced changes in GF models of AMD; future studies, including laser-induced CNV and other AMD animal models and aging studies, should be directed to assess potential relationships between HFDs and the pathogenesis of retinal disease, as well as to explore the dietary effects in the absence of gut microbiota. Additionally, emerging evidence suggests a more granular approach towards dietary fat and retinal disease may be warranted, as specific types of fat, such as saturated fats and oleic acid, may confer different risk profiles for AMD [6,146,147,148].

AMD is a multifactorial, vision-threatening disease whose prevalence is expected to increase dramatically as other factors, including obesity, diabetes, and average population age, continue to rise [1,149]. Diet is one of the primary modifiable risk factors for AMD progression. However, interventions targeting AMD through diet are rare. Current examples, such as AREDS nutritional supplementation, have limited efficacy and are found to reduce the risk of AMD progression in only certain sub-populations [150,151]. Therefore, investigating the complex interactions between diet, the gut microbiome, and retinal health may potentially unlock new insights for AMD prevention, as well as develop the foundation for interventions that are minimally invasive and cost-effective.

## Figures and Tables

**Table 1:** Top 30 differentially expressed genes upregulated by high-fat diet (HFD).

Gene	LogFC	Adjusted *p*-Value	Protein
*Cd244a*	6.27	1.62 × 10^−3^	Natural Killer Cell Receptor 2B4
*Ripply1*	5.52	9.98 × 10^−3^	Ripply Transcriptional Repressor 1
*Lilrb4a*	5.28	4.81 × 10^−2^	Leukocyte Immunoglobulin-Like Receptor Subfamily B member 4
*Fcer1a*	5.26	2.85 × 10^−2^	Fc Epsilon Receptor Ia
*Dnajc22*	5.24	1.44 × 10^−2^	DnaJ Heat Shock Protein Family (Hsp40) Member C22
*Alkal2*	5.11	3.11 × 10^−2^	ALK And LTK Ligand 2
*Ncr1*	5.04	1.16 × 10^−2^	Natural Cytotoxicity Triggering Receptor 1
*Ccl19*	4.94	7.60 × 10^−3^	C-C Motif Chemokine Ligand 19
*Slc38a11*	4.81	3.85 × 10^−2^	Solute Carrier Family 38 Member 11
*Ces2e*	4.71	4.62 × 10^−2^	Pyrethroid Hydrolase Ces2e
*Lpar3*	4.66	1.73 × 10^−2^	Lysophosphatidic Acid Receptor 3
*Sele*	4.62	4.49 × 10^−2^	Selectin E
*Sirpb1a*	4.61	9.02 × 10^−3^	Signal-Regulatory Protein Beta 1A
*Efhb*	4.57	1.33 × 10^−2^	EF-Hand Domain-Containing Family Member B
*Pgpep1l*	4.56	2.15 × 10^−2^	Pyroglutamyl-Peptidase 1-Like Protein
*Tnfrsf13b*	4.54	2.94 × 10^−2^	Tumor Necrosis Factor Receptor Superfamily Member 13B
*Il12b*	4.49	2.65 × 10^−2^	Interleukin-12 Subunit Beta
*Tmem232*	4.46	2.53 × 10^−2^	Transmembrane Protein 232
*Trbc1*	4.41	1.51 × 10^−2^	T Cell Receptor Beta Constant 1
*Slc4a1*	4.34	3.36 × 10^−2^	Solute Carrier Family 4 Member 1
*Olfr574*	4.34	1.60 × 10^−2^	Olfactory Receptor Family 51 Subfamily T Member 1
*Xlr*	4.28	5.51 × 10^−3^	X-Linked Lymphocyte-Regulated Protein PM1
*Gpr141*	4.25	4.73 × 10^−2^	G Protein-Coupled Receptor 141
*Cnr2*	4.25	3.48 × 10^−2^	Cannabinoid Receptor 2
*Mael*	4.23	4.08 × 10^−2^	Maelstrom Spermatogenic Transposon Silencer
*Lao1*	4.22	4.06 × 10^−2^	Amine Oxidase
*Mcoln2*	4.16	1.72 × 10^−2^	Mucolipin TRP Cation Channel 2
*Ccl22*	4.16	3.77 × 10^−2^	C-C Motif Chemokine Ligand 22
*Rnase1*	4.11	2.66 × 10^−2^	Ribonuclease A Family Member 1, Pancreatic
*Ptgs2os*	4.11	3.37 × 10^−2^	Prostaglandin-Endoperoxide Synthase 2, Opposite Strand

**Table 2:** Top 10 biological processes upregulated by high-fat diet (HFD).

Biological Processes	*p*-Value	Adjusted *p*-Value	Gene Ratio	Genes
Melanocyte differentiation	1.79 × 10^−7^	9.49 × 10^−5^	0.35	*Edn3*, *Ednrb*, *Mitf*, *Mlph*, *Rab27a*, *Slc24a5*, *Sox10*, and *Tyrp1*
Angiogenesis	2.08 × 10^−13^	5.51 × 10^−10^	0.13	*Ackr3*, *Angpt1*, *Angpt2*, *Angptl2*, *Apold1*, *Calcrl*, *Cfh*, *Clic4*, *Col18a1*, *Col8a1*, *Col8a2*, *Cxcr3*, *Cyp1b1*, *Ecscr*, *Ephb4*, *Esm1*, *Fap*, *Fzd8*, *Htatip2*, *Mcam*, *Nrp2*, *Pik3r6*, *Plxnd1*, *Ptgs2*, *Ptprb*, *Rapgef3*, *Rhoj*, *Rspo3*, *Tbx4*, *Tek*, *Tgfbr3*, *Tie1*, *Tnfaip2*, and *Vegfc*
Cell surface receptor signaling pathway	1.50 × 10^−9^	1.50 × 10^−6^	0.13	*Adgra3*, *Adgrf5*, *Adgrg6*, *Calcrl*, *Cd22*, *Cd86*, *Cxcr3*, *Cysltr1*, *Edn3*, *Fcer1a*, *Fzd2*, *Fzd4*, *Fzd7*, *Fzd8*, *Gpr157*, *Il12b*, *Itgal*, *Itpkb*, *Npr1*, *Osmr*, *Ostn*, *Pth1r*, *Tnfrsf1b*, and *Tshr*
Positive regulation of angiogenesis	5.68 × 10^−7^	2.15 × 10^−4^	0.12	*Angpt2*, *Brca1*, *Chil1*, *Cxcr3*, *Cybb*, *Cyp1b1*, *Cysltr1*, *Ets1*, *Itgb3*, *Itgb8*, *Pik3r6*, *Ptgis*, *Rapgef3*, *Tek*, *Tgfbr2*, *Thbs1*, *Tie1*, and *Vegfc*
Inflammatory response	1.14 × 10^−9^	1.50 × 10^−6^	0.10	*Agtr1a*, *Axl*, *Bmp2*, *Bmp6*, *Ccl19*, *Ccl22*, *Ccl4*, *Cfh*, *Chil1*, *Cnr2*, *Csf1r*, *Cxcl10*, *Cxcr3*, *Cyba*, *Cybb*, *Cysltr1*, *Gbp5*, *Il25*, *Lilrb4a*, *Lipa*, *Ly86*, *P2rx7*, *Pla2g2e*, *Prkcq*, *Ptgs1*, *Ptgs2*, *Rarres2*, *Sele*, *Selp*, *Slc11a1*, *Thbs1*, *Themis2*, *Tlr13*, and *Tnfrsf1b*
Response to bacterium	1.29 × 10^−6^	3.56 × 10^−4^	0.09	*Adamts9*, *Bank1*, *Bmp2*, *Cxcl10*, *Fkbp5*, *Gbp5*, *Gpc3*, *Gzma*, *Ifi211*, *Ifit3*, *Iigp1*, *Lrat*, *Ms4a1*, *Myo1f*, *Naaladl2*, *Nexn*, *Ociad2*, *P2rx7*, *Rnase1*, *Serpina3f*, *Serpinb9*, *Slc11a1*, *Tgtp1*, and *Trf*
Immune response	8.75 × 10^−7^	2.90 × 10^−4^	0.08	*Ackr3*, *Azgp1*, *B2m*, *Bmp6*, *Ccl19*, *Ccl22*, *Ccl4*, *Cd28*, *Cd86*, *Cfh*, *Colec12*, *Ctsk*, *Ctss*, *Cxcl10*, *Cxcl12*, *Cxcr3*, *Endou*, *Enpp2*, *H2-Ab1*, *H2-Eb1*, *H2-M3*, *Itgb8*, *Ly86*, *Serpinb9*, *Tgfbr3*, *Tgtp1*, *Tnfrsf1b*, *Tnfsf10*, *Tnfsf14*, *Vav1*
Cell adhesion	1.48 × 10^−8^	9.80 × 10^−6^	0.08	*Ackr3*, *Azgp1*, *Cd22*, *Cd33*, *Cd84*, *Cldn1*, *Cldn2*, *Cntnap4*, *Col12a1*, *Col18a1*, *Col8a1*, *Col8a2*, *Cyp1b1*, *Dpp4*, *Ephb4*, *Fap*, *Fblim1*, *Fbln5*, *Gpnmb*, *Hpse*, *Icam2*, *Itga9*, *Itgal*, *Itgb3*, *Itgb8*, *Jcad*, *Kitl*, *Lgals3bp*, *Ly9*, *Mcam*, *Mybpc2*, *Nid2*, *Pcdh12*, *Plpp3*, *Sele*, *Sell*, *Selp*, *Siglecf*, *Spp1*, *Svep1*, *Thbs1*, *Vcam1*, and *Vwf*
Positive regulation of cell population proliferation	1.98 × 10^−7^	9.49 × 10^−5^	0.07	*Adora2b*, *Agtr1a*, *Aldh1a2*, *Bambi*, *Calcrl*, *Cd38*, *Cdk2*, *Clec7a*, *Col18a1*, *Csf1r*, *Cxcl10*, *Cxcl12*, *Cxcr3*, *Dpp4*, *Edn3*, *Ednra*, *Ednrb*, *Enpp2*, *Esm1*, *Ets1*, *Fgf7*, *Gab2*, *Gcnt2*, *Gli1*, *Kitl*, *Lrp5*, *Nog*, *Ntn1*, *Osmr*, *Pax3*, *Pdgfc*, *Pdgfd*, *Ptgs2*, *Pth1r*, *S100b*, *Stox1*, *Tgfbr3*, *Thbs1*, *Tshr*, and *Vegfc*
Multicellular organism development	1.21 × 10^−6^	3.56 × 10^−4^	0.06	*Ackr3*, *Angpt1*, *Angpt2*, *Ano1*, *Axl*, *B2m*, *Bmp2*, *Bmp6*, *Cdh19*, *Csf1r*, *Ecscr*, *Eda2r*, *Ephb4*, *Eya1*, *Eya2*, *Fhl1*, *Foxd1*, *Foxd3*, *Fst*, *Fzd2*, *Fzd4*, *Fzd7*, *Fzd8*, *Gli1*, *Gpr157*, *Gsx2*, *Htatip2*, *Krt8*, *Lbx1*, *Lrp5*, *Mael*, *Mertk*, *Met*, *Mgp*, *Mitf*, *Nog*, *Nrp2*, *Ostn*, *Pax3*, *Pdgfc*, *Pdgfd*, *Pitx2*, *Plpp3*, *Plxnd1*, *Ripply1*, *Sema3b*, *Sema3c*, *Sema6d*, *Serpine2*, *Sfrp5*, *Shisa2*, *Smoc1*, *Sox6*, *Stpg4*, *Tbx4*, *Tek*, *Tie1*, *Tmem88*, *Tnfaip2*, *Vegfc*, and *Wipf3*

**Table 3:** Top 10 molecular pathways upregulated by high-fat diet (HFD).

Molecular Pathways	*p*-Value	Adjusted *p*-Value	Gene Ratio	Genes
Hemoglobin binding	2.43 × 10^−5^	3.96 × 10^−3^	0.57	*Hbb-bs*, *Hbb-bt*, *Lrp2*, *Slc4a1*
Sialic acid binding	2.30 × 10^−5^	3.96 × 10^−3^	0.38	*Cd22*, *Cd33*, *Sele*, *Selp*, *Siglecf*
Extracellular matrix binding	2.09 × 10^−5^	3.96 × 10^−3^	0.23	*Adamts15*, *Clec14a*, *Dcn*, *Itgb3*, *Smoc1*, *Spp1*, and *Thbs1*
Fibronectin binding	2.64 × 10^−5^	3.96 × 10^−3^	0.23	*Ctsk*, *Ctss*, *Fbln1*, *Igfbp3*, *Igfbp5*, *Itgb3*, *Thbs1*
Integrin binding	2.81 × 10^−7^	2.12 × 10^−4^	0.13	*Cxcl12*, *Esm1*, *Fap*, *Fbln1*, *Fbln5*, *Fbn1*, *Gpnmb*, *Icam2*, *Itgb3*, *Itgb8*, *Lcp1*, *Lilrb4a*, *Plpp3*, *Spp1*, *Thbs1*, *Vcam1*, and *Vwf*
Extracellular matrix structural constituent	5.40 × 10^−5^	4.08 × 10^−3^	0.11	*Col10a1*, *Col18a1*, *Col8a1*, *Col8a2*, *Col9a3*, *Fbln1*, *Fbn1*, *Fbn2*, *Matn2*, *Nid2*, *Ntn1*, *Thbs1*, *Vwf*
Signaling receptor activity	4.79 × 10^−6^	1.21 × 10^−3^	0.10	*Cd48*, *Colec12*, *Cxcr3*, *Eda2r*, *Fzd4*, *Itgb8*, *Klrk1*, *Lrp2*, *Mrc2*, *P2rx7*, *Paqr6*, *Stra6*, *Tek*, *Tgfbr2*, *Tlr13*, *Tnfrsf19*, *Trem2*, and *Tshr*
Carbohydrate binding	2.04 × 10^−6^	7.73 × 10^−4^	0.10	*Agl*, *C4b*, *Cd22*, *Cd33*, *Clec12a*, *Clec14a*, *Clec1a*, *Clec4a2*, *Clec4d*, *Clec4n*, *Colec12*, *Galm*, *Galnt6*, *Klrk1*, *Man2a1*, *Mrc2*, *Sele*, *Sell*, *Selp*, *Siglecf*
Heparin binding	4.12 × 10^−5^	3.96 × 10^−3^	0.10	*Adamts1*, *Adamts15*, *Apoe*, *Cfh*, *Cxcl10*, *Fbn1*, *Fgf7*, *Gpnmb*, *Nrp2*, *Rspo3*, *Selp*, *Serpine2*, *Smoc1*, *Tgfbr3*, and *Thbs1*
Protein homodimerization activity	2.24 × 10^−5^	3.96 × 10^−3^	0.06	*Ano1*, *Ano6*, *Apoe*, *Atp2a1*, *B2m*, *Cat*, *Csf1r*, *Dpp4*, *Dpyd*, *Fap*, *Fbln5*, *Fzd4*, *Galm*, *Gbp3*, *Gbp5*, *Gzma*, *H2-M3*, *Hnf4a*, *Impa2*, *Man2a1*, *Mgll*, *Nog*, *Npr3*, *Pdgfc*, *Pitx2*, *Pon1*, *Pon3*, *Ptgs2*, *Pth1r*, *Rdh5*, *S100b*, *Slc11a1*, *Slc4a1*, *St6gal1*, *Tpd52l1*, *Trim21*, *Trim30d*, *Tyr*, *Tyrobp*, and *Tyrp1*

## Data Availability

The complete dataset of identified DEGs is available in Supplementary Table S1.

## Supplementary Materials

The following are available online at https://www.mdpi.com/article/10.3390/cells11132076/s1, Table S1: provides all 649 identified DEGs.
